# Betaine Inhibits NLRP3 Inflammasome Hyperactivation and Regulates Microglial M1/M2 Phenotypic Differentiation, Thereby Attenuating Lipopolysaccharide-Induced Depression-Like Behavior

**DOI:** 10.1155/2022/9313436

**Published:** 2022-10-26

**Authors:** Man Zhang, Xiao-Long Wang, Hui Shi, Lan-Qing Meng, Hong-Feng Quan, Lin Yan, Hui-Fang Yang, Xiao-Dong Peng

**Affiliations:** ^1^Department of Basic Pharmacology and Toxicology, School of Pharmacy, Ningxia Medical University, Yinchuan, China; ^2^Ningxia Chinese Medicine Research Center, Yinchuan, China; ^3^Ningxia Collaborative Innovation Center of Regional Characteristic Traditional Chinese Medicine, Ningxia Medical University, Yinchuan, Ningxia, China; ^4^Key Laboratory of Fertility Preservation and Maintenance of Ministry of Education, School of Basic Medical Sciences, Ningxia Medical University, Yinchuan, China; ^5^School of Public Healthy and Management, Key Laboratory of Environmental Factors and Chronic Disease Control, Ningxia Medical University, Yinchuan, China

## Abstract

Depression is one of the most important mental illnesses and is closely related to inflammation. Betaine is a natural product with an anti-inflammatory and antioxidant activities. However, the mechanism by which betaine ameliorates depression-like behaviors induced by lipopolysaccharide (LPS) is poorly understood. The purpose of this study was to investigate the neuroprotective effect of betaine on LPS-induced depression-like behavior in mice and its mechanism of action. ICR mice were randomly divided into four groups: the control group, the LPS model group (0.83 mg/kg), the positive drug group (MIDO, 50 mg/kg), and the betaine group (5% and 1% in drinking water). The betaine group was administered for 21 days, and on the 22nd day, except for the blank group, LPS (0.83 mg/kg) was intraperitoneally injected to establish a lipopolysaccharide-induced mice depression-like model. Twenty-four hours after LPS injection, the tail suspension test (TST), open field test (OFT), and sucrose preference test (SPT) were performed to evaluate the effect of betaine on LPS-induced depressive behavior in mice. After the behavioral study, the mouse brain, hippocampus, and serum were taken for detection. The expressions of cytokines and inflammatory mediators were detected by ELISA, HE staining, immunofluorescence, immunohistochemistry, and western blotting. Western blotting was used to detect the protein expression levels of the nucleotide-binding oligomerization domain-like receptor protein 3 (NLRP3), caspase-1, and ASC, the protein expression levels of the microglial polarization markers COX-2, inducible nitric oxide synthase (iNOS), and CD206. The results showed that betaine significantly ameliorated the depression-like behavior in LPS-induced mice, significantly attenuated the production of proinflammatory cytokines and increased the release of an anti-inflammatory cytokines. Betaine decreased the expression of the NLRP3 inflammasome, decreased the expression of M1 polarization markers, tumor necrosis factor-alpha (TNF-*α*), interleukin-1*β* (IL-1*β*), COX-2, and iNOS and promoted the expression of M2 polarization marker CD206. Our study suggests that betaine may promote the transition of microglia from the M1 to the M2 phenotype by inhibiting NLRP3 inflammasome activation, thereby attenuating lipopolysaccharide-induced depression-like behavior.

## 1. Introduction

Depression, as a mental illness, has many symptoms, such as cognitive impairment, loss of appetite, pessimism, and suicidal tendencies. The inflammatory hypothesis of depression was proposed in 1991 and attracted widespread attention [1], and the neuroimmunoinflammatory hypothesis of depression was proposed in 1995 [2]. Since then, the relationship between inflammatory cytokines and depression has been increasingly studied. Exploring the innate immune function of glial cells is critical to understanding the role of the brain's immune system in fighting inflammation and psychiatric disorders.

Excessive production of inflammatory cytokines and microglial activation in the brain signals depression and dysfunction in brain signaling. Microglia belong to the nonglial system and are resident mononuclear phagocytes in the central nervous system [3]. During the central nervous system injury from infection, traumatic brain injury, or ischemic injury, microglia change from a quiescent state to an amoeba-activated state. Activated microglia can be divided into two main types: classical- (proinflammatory, M1) and alternative- (anti-inflammatory, M2) activated states. Proinflammatory cytokines, interferons, tumor necrosis factor (TNF), and lipopolysaccharide (LPS) can often activate microglia [4]. Depression can induce the polarization of microglia into the M1 to secrete proinflammatory factors. Various antidepressant drugs have an anti-inflammatory effects and can reverse the polarization of microglia to the M1 type. The M2 type can improve symptoms related to depression [5]. However, few pharmacological compounds have been shown to modulate the transformation of microglia to the M2 phenotype [6, 7]. Therefore, the drug-induced polarization of microglia from the M1 phenotype to the M2 phenotype may provide a new strategy for the treatment of depression.

Environmental stress before the onset of microglia-dependent depression increases the steady-state concentration of the activated NLRP3 inflammasome, and the activity of the NLRP3 inflammasome and its tight regulation largely determines the morphology and the regulation of microglia. Studies have found that NLPR3 gene knockout (NLRP3−/−) [8] or Caspase-1 gene knockout (CASP1−/−) [9] can alleviate depressive-like behaviors in mice due to chronic stress and NLRP3−/−; the microglial morphology in mice is biased toward an M2-like state [10]. The study found that NLRP3 inhibitors had inhibitory effects on LPS-induced depression in a mouse model, showing the key role of the NLPR3 inflammasome in linking stress and neuroinflammatory states.

The NLRP3 inflammasome has been shown to be activated in patients with depression [11]. Knockout of NLRP3 in mice alleviated depression-like behaviors and suppressed proinflammatory processes [12]. Studies have shown that in CUMS-induced depression, NLRP3 polarizes microglia toward the M1 phenotype [13]. It has recently been reported that patients with major depressive disorder have higher levels of NLRP3, caspase-1, and IL-1*β* in serum or peripheral blood mononuclear cells [14]. Furthermore, when caspase-1 was specifically inhibited, this led to a decrease in depression-like behaviors triggered by multiple stimuli, such as estrogen deficiency, chronic mild stress, and LPS injection [14]. To be precise, NLRP3 and caspase-1 are closely related to depression, which lays the foundation for further research. Therefore, modulating the NLRP3 signaling pathway and further inhibiting neuroinflammation may be a therapeutic strategy to improve depression.

Minocycline (MIDO), a broad-spectrum tetracycline antibiotic, has various beneficial effects on the central nervous system, including anti-inflammatory, antioxidant, and neuroprotective effects [15, 16]. Various studies have demonstrated that minocycline is considered a novel treatment for several psychiatric disorders associated with inflammation, including depression [16]. Various antidepressant drugs have an anti-inflammatory effects and can reverse the polarization of microglia to the M1 type. Therefore, we used minocycline as a positive control.

**Figure 1:**
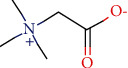
Chemical structure of betaine.

The long-term use of western medicine in the treatment of depression may cause adverse reactions such as dependence, toxicity, memory loss, organ dysfunction, and delayed clinical effects, while herbal medicine for depression has made great progress in delaying the course of the disease, improving the efficacy of western medicine, and reducing side effects. Herbal medicines with antidepressant effects (herbal antidepressants), including prescriptions, individual herbs, and phytochemicals, are also widely used to treat depression. [17, 18]. Betaine (Figure 1), also known as trimethyl glycine, is a water-soluble compound that penetrates the blood-brain barrier and is highly distributed in the hippocampus in vitro [19, 20]. Betaine is mainly used as an osmotic regulator and a methyl donor to participate in the dual physiological role of metabolism in vivo [21]. Betaine has various pharmacological effects, such as antiepileptic, neuroprotective, and memory-improving effects [19, 22, 23]. In particular, it acts as a methyl donor to interfere with depression caused by the hyperhomocysteinemia [24]. However, whether betaine inhibits lipopolysaccharide-induced microglial phenotype polarization remains unclear. In our previous study, it was found that betaine can regulate the phenotypic changes of microglia, thereby reducing the inflammatory response of microglial cells induced by LPS stimulation, and the effect of betaine is related to the regulation of the nuclear factor-*κ*B (NF-*κ*B) signaling pathway [25]. However, the effect of betaine on LPS-induced depression-like behavior has not been fully elucidated. Importantly, the polarization of the microglial phenotype and the role of inflammatory markers in response to betaine treatment have not been well-studied. Therefore, the purpose of this study was not only to investigate the role of betaine in response to the LPS model of depression but also to explore the mechanism of its role in microglial polarization.

## 2. Materials and Methods

### 2.1. Animals

Sixty male, six-week-old ICR mice, weighing 18–22 g, were purchased from the Animal Center of Ningxia Medical University. The batch number is SCXK (Ning) 2021-0001. All animals were housed under standard conditions with a temperature of 23 ± 5°C, a dark/light cycle of 12 h/12 h, and a humidity of 40–60%. These animals had free access to food and water. This study was approved by the Animal Experiment Ethics Committee of Ningxia Medical University.

### 2.2. Animal Experimental Design

Mice were randomly divided into the control group (*n* = 12), the LPS model group (0.83 mg/kg, *n* = 12), the positive drug group (MIDO, 50 mg/kg, *n* = 12), the 5% betaine group (*n* = 12), and the 1% betaine group (*n* = 12). Betaine (purity >99%, B2629, Sigma-Aldrich) at concentrations of 5% and 1% was added to the drinking water of mice, and minocycline (MIDO, 50 mg/kg, i.p.) was used as a positive control. LPS (L2630, Sigma-Aldrich) and MIDO (M9511, Sigma-Aldrich) were dissolved in 0.9% (*w*/*v*) saline. The dose that gave the best efficacy in the previous preexperiment was 5% betaine. Each group was given the corresponding vehicle and drugs for 21 consecutive days. There was no difference in drinking water consumption between the animals exposed to betaine and the tap water controls. On the 15th day, the positive drug group was given an intraperitoneal injection of MIDO. At 9:00 in the morning of the 22nd day, except for the normal group, LPS (0.83 mg/kg) was intraperitoneally injected, and the body weight and food intake were monitored in the following two hours, six hours, ten hours, and twenty-four hours, and voluntary activities were observed at two hours and twenty-four hours. After the injection of LPS, the sugar water experiment was performed, and 24 hours after the injection, the behavioral test was performed. Blood was taken after the behavioral test, and the whole brain and hippocampus were collected. Brains were fixed with 4% paraformaldehyde (P0099, Beyotime Biotechnology, China) or store in -80°C low temperature freezer (Thermo Scientific Forma902, Thermo Fisher Instruments Co., Ltd.) until use.

## 3. Behavior Tests

### 3.1. Mouse Food Intake and Body Weight Measurement

Before the injection of LPS, each group of mice was tested for body weight, given food, and weighed. At 24 hours after the injection of LPS, the body weight and food intake of each group of mice were tested, and the difference in weight was calculated.

### 3.2. Sucrose Preference Test (SPT)

The mice were trained to adapt to two bottles of 1% sucrose solution (*w*/*v*) for 24 h, followed by two bottles of pure water for 24 h. After the deprivation of food and water for 12 h, every mouse was individually faced with two bottles filled with 1% sucrose solution or pure water for 24 h. The feeder locations were switched at 12 h intervals to avoid possible site preference. Consequently, the consumption of sucrose solution and pure water was assayed. Sucrose preference was calculated according to the formula: sucrose preference (%) = sucrose consumption (g)/[sucrose consumption (g) + pure water consumption (g)] × 100%.

### 3.3. Tail Suspension Test (TST)

In a quiet, dark room, the mice were vertically suspended in a 50 cm × 50 cm × 50 cm open box without lids, with the mouse nose tip at about 5 cm from the bottom of the carton box and connected to a JH-2 tension transducer. After the start of suspension, the force changes of the tension transducer were recorded by the BL-420E+ biological function experimental system for 6 minutes, and the immobility time of the mice was counted after 4 minutes.

### 3.4. Open Field Test (OFT)

The OFT was carried out with a square arena apparatus (50 × 50 × 40 cm) with 16 equal squares. Each mouse was individually placed in the central area facing the wall. The behavior of the mice was observed over the next 5 minutes. The distance traveled and time spent in the central area were monitored.

## 4. Methods

### 4.1. The Level of TNF-*α*, IL-6, IL-1*β*, IL-18, and IL-10 in the Mouse Hippocampus Were Detected with Kits (Jiang Lai Biological Co., Ltd.)

TNF-*α*, IL-6, IL-1*β*, IL-18, and IL-10 in the mice hippocampus were detected with kits (Jiang Lai Biological Co., Ltd.). Tissue corticosterone and C-reactive protein levels were detected with kits (Jiang Lai Biological Co., Ltd.). The hippocampi were weighed and chopped. The samples were treated with ice-cold PBS containing protease inhibitor and then centrifuged at 4000 g at 4°C (refrigerated high-speed centrifuge, Fresco 17, Thermo Fisher). The supernatant was subjected to ELISA measurement. All ELISA experiments were conducted in accordance with the manufacturer's protocol. The absorbance was calculated by standard curves.

### 4.2. HE Staining

Intact brain tissue was removed and dehydrated with different concentrations of ethanol (dehydrator, JJ-12J, Wuhan Junjie Electronics Co., Ltd.). Immerse the dehydrated brain tissue in paraffin at 60°C for sectioning and take coronal sections (microtome, RM2016, Shanghai Leica Instrument Co., Ltd.). Sections were then incubated with hematoxylin and eosin staining solution for 5 min. Dehydrate with xylene (10023418, Sinopharm Chemical Reagent Co., Ltd.) and add neutral glue to seal the slices. The pathological changes of brain tissue were observed under light microscope (XSP-C204, CIC).

### 4.3. Immunohistochemistry

The brain tissue of each group of mice was perfused with normal saline, perfused with paraformaldehyde, and placed into a fixative. The primary antibodies used were COX-2 (1 : 500, GB11077-1, Servicebio, China) and CD206 (1 : 500, GB113497, Servicebio, China). Brain coronal sections (microtome, RM2016, Shanghai Leica Instrument Co., Ltd.) were washed 3 times with PBS for 5 min each, placed in citrate buffer (pH 6.0, G1202, Servicebio, China), and then placed in a microwave oven to start antigen retrieval. After antigen retrieval, equilibrate to room temperature and wash 3 times with PBS for 5 min each. Incubate in 3% hydrogen peroxide solution for 20 minutes, then begin a total of 3 washes of 5 minutes each. After blocking with 5% goat serum (G1209, Servicebio, China) for 1 h, the blocking solution was discarded. The corresponding primary antibody solution was added and incubated with the samples at 4°C overnight. The next day, after equilibrating at room temperature, the primary antibody solution was aspirated, and the samples were washed three times with PBS for 5 min each time. The required secondary antibody (G1213, Servicebio, China) was added dropwise and the samples were placed at room temperature for approximately 2 h. The secondary antibody solution was then poured off, and the samples were washed three times in PBS for 5 min each time and incubated with avidin-coupled horseradish peroxidase complex solution at room temperature for 1 h. The avidin-coupled horseradish peroxidase complex solution was discarded, and samples were washed with PBS three times for 5 min each time. DBA (G1211, Servicebio, China) chromogenic solution was added, dropwise, color was developed for 20-60 s, and the samples were washed with PBS. They were then dehydrated with gradient ethanol, cleared with xylene, sealed with neutral gum, and observed under a microscope (XSP-C204, CIC).

### 4.4. Immunofluorescence

After the behavioral test, the brains were fixed with 4% paraformaldehyde (P0099, Beyotime Biotechnology, China) and dehydrated. Brain coronal slices of 10 *μ*m were made with a microtome (RM2016, Shanghai Leica Instruments Co., Ltd.). Sections were washed and blocked with 0.3% Triton and 5% calf serum (F8687, Beyotime Biotechnology, China) for 1.5 hours at room temperature and then incubated with primary antibodies detecting Iba1 (1 : 6000, GB13105-1, Servicebio, Wuhan, China) and NLRP3 (1 : 3000, DF7438, Servicebio, Wuhan, China) in the dark at 4°C for 24 hours in medium, followed by incubation with secondary antibody (G1213, Servicebio, China) for 1 hour at room temperature. The nuclei were counterstained with DAPI (G1012, Servicebio, China), and the sections were slightly dried and mounted with an antifluorescence quenching mounting medium. Sections were observed under a fluorescence microscope, and images were collected. Images were taken under a microscope (Nikon Eclipse C1, Nikon, Japan). To quantify phenotypic changes in microglia, the proportional areas were analyzed with Iba1 labeling and NLRP3 staining as previously described. The proportional area is shown as the average area of the positive labels for all the representative pictures.

### 4.5. Western Blot Analysis

Mice were sacrificed after behavioral testing. Under sterile conditions, hippocampal tissue was rapidly removed from each mouse brain and placed in liquid nitrogen. Tissue samples were homogenized in radioimmunoprecipitation assay lysis buffer (RIPA lysis buffer, G2002, Servicebio), then centrifuged at 12,000× g for 15 min (grinder, KZ-II, Shanghai Jingxin Industrial Development Co., Ltd.). The supernatant was then collected at 4°C. The BCA (KGPBCA, KeyGEN BioTECH, China) protein assay and western blot were performed to determine protein concentration. Western blot analysis was used to detect protein expression in the cells, using antibodies against the following proteins: CD206 (1 : 1000), iNOS (1 : 1000), COX-2 (1 : 1000), NLRP3 (1 : 1000), P-NF-*κ*B (1 : 500), and *β*-actin (1 : 2000, Proteintech, Wuhan, China); TLR4 (1 : 500), Myd88 (1 : 1000), ASC (1 : 1000), and Caspase-1 (1 : 1000) (Abcam, Cambridge, MA, USA). This was followed by treatment with HRP-anti-rabbit secondary antibody (1 : 10,000; ProteinTech, Wuhan, China) for 1.5 h at 20°C. An ECL agent (Thermo Fisher Scientific, Inc., Waltham, MA, USA) was used to analyze the protein bands, and the ImageJ software (NIH, Bethesda, MD, USA) was used to analyze protein expression.

### 4.6. Statistical Analysis

All values were expressed as mean ± SEM and analyzed using SPSS Statistics V22.0 (IBM, Armonk, NY, USA). The data is normally distributed (normality test). Data were statistically analyzed using one-way analysis, followed by Student's *t*-test (comparing two groups) or LSD post hoc test (comparing more than two groups). *P* < 0.05 was considered statistically significant.

## 5. Results

### 5.1. Effects of Betaine on LPS-Induced Depression-Like Behavior

**Figure 2:**
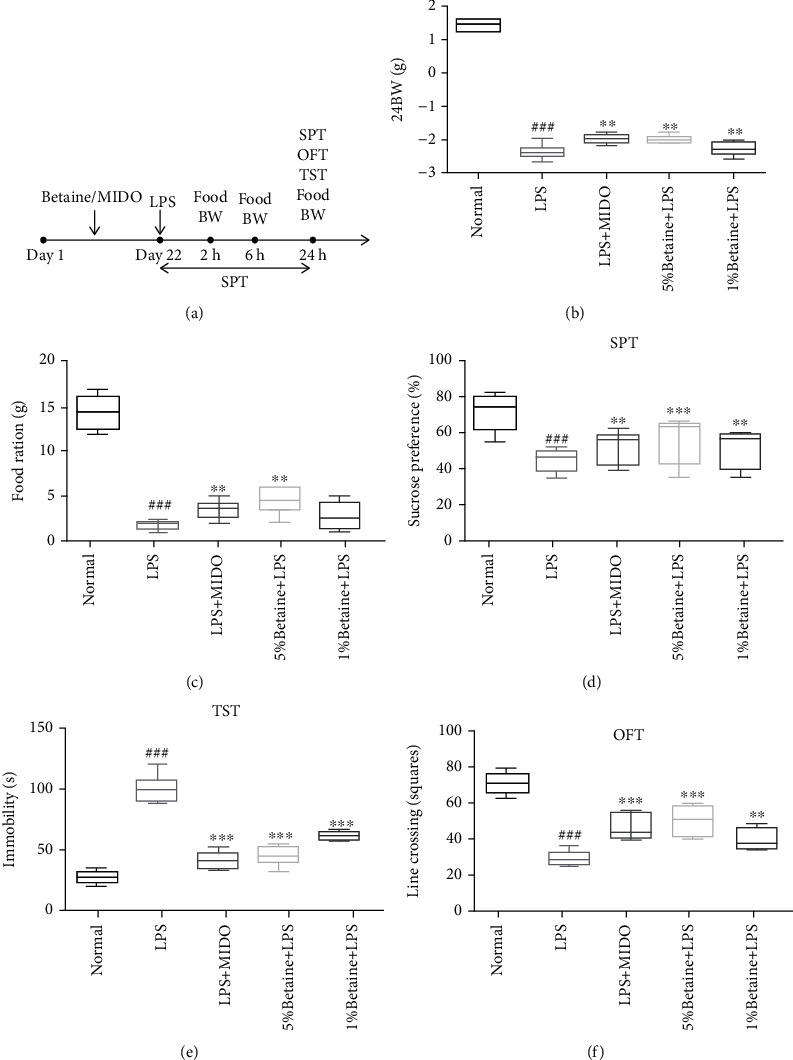
Effects of betaine on food intake, body weight, and depressive behavior in LPS-induced depression-like mice. (a) Experimental flow chart. (b) Changes in body weight of mice, 24 hours after LPS injection. (c) Changes in food intake in mice, 24 hours after LPS injection. (d) Influence of betaine on sucrose preference test. (e) Influence of betaine on tail suspension test. (f) Influence of betaine on open field test. The results were expressed as median ± interquartile range; ^###^*P* < 0.01 compared to the normal control group; ^∗∗^*P* < 0.01 and ^∗∗∗^*P* < 0.001 compared to the model group, *n* = 12.

Behavioral assays to evaluate the effects of betaine on the LPS-induced mice (Figure 2(a)). The results showed that betaine could significantly improve the feeding behavior and decreased food intake (Figure 2(c)) and weight loss (Figure 2(b)) of LPS-induced model mice. Betaine significantly improved the autonomous behavior of LPS-induced model mice (Figure 2(f)), significantly shortened the immobility time of mice in the tail suspension experiment (Figure 2(e)), and significantly increased the preference of LPS-induced model mice for sucrose drinking (Figure 2(d)).

### 5.2. Effects of Betaine on the Morphological Changes in Pyramidal Cells in the CA1 Region of the Hippocampus of Mice Induced by LPS

**Figure 3:**
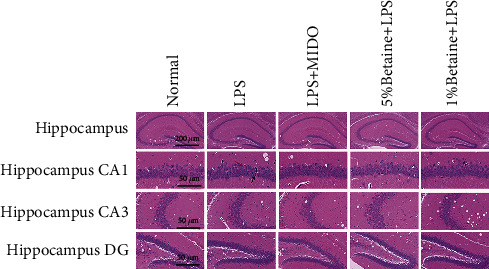
HE staining to observe the morphological changes of LPS-induced mice hippocampal CA1, CA3, and DG regions, *n* = 6. Red arrows indicate the irregular arrangement of neurons with enlarged pericellular spaces, black arrows indicate the neuronal pyknosis and the deepened coloration.

Betaine significantly improved the integrity of cellular morphological features in the hippocampal brain region of mice with LPS-induced depression-like behavior. The cells in the hippocampus of mice in the normal group were neatly arranged; in the model group, the cells in the hippocampus were irregularly arranged; some neurons had pyknotic nuclei and a small number of microglia phagocytosed necrotic neurons. Even light red plaques can be seen in some areas (Figure 3). Both betaine and minocycline improved the damage to hippocampal neurons to different degrees. The results suggest that betaine has a protective effect on the histomorphological damage of neurons in the hippocampus of mice with acute LPS-induced depression-like behavior.

### 5.3. The Effects of Betaine on the Content of Inflammatory Factors in the Hippocampus of Mice

**Figure 4:**
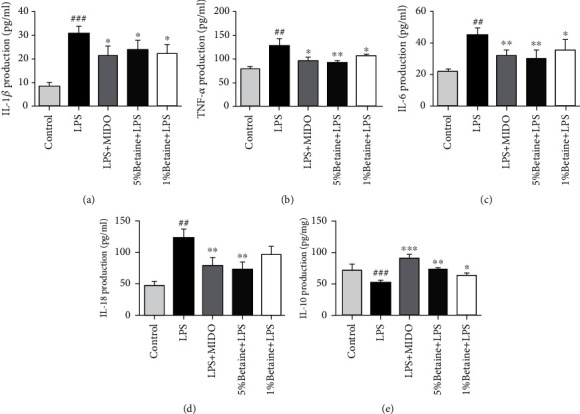
Effects of betaine on LPS-induced inflammatory cytokine production in mice hippocampus. (a–e) The expression levels of IL-1*β*, IL-6, IL18, TNF-*α*, and IL-10 in mouse hippocampus were determined by ELISA, with minocycline as the positive control. The results were expressed as mean ± SEM; ^##^*P* < 0.01 and ^###^*P* < 0.001 compared to the normal control group; ^∗^*P* < 0.05 and ^∗∗^*P* < 0.01 compared to the model group, *n* = 4.

LPS stimulation significantly increased the levels of M1 proinflammatory cytokines, including TNF-*α*, IL-1*β*, and IL-6 and decreased the level of the M2 anti-inflammatory cytokine IL-10. Compared with the model group, betaine significantly reduced the levels of the IL-1*β*, IL-6, IL-18, and TNF-*α* inflammatory factors and increased the amount of IL-10 in the hippocampus of LPS-treated mice (Figures 4(a)–4(e)). The results suggest that betaine can inhibit the production of proinflammatory factors in the brains of mice induced by LPS while promoting the production of anti-inflammatory factors.

### 5.4. Effects of Betaine on Serum C-Reactive Protein (CRP) and Corticosterone (CORT) Levels in Mice

**Figure 5:**
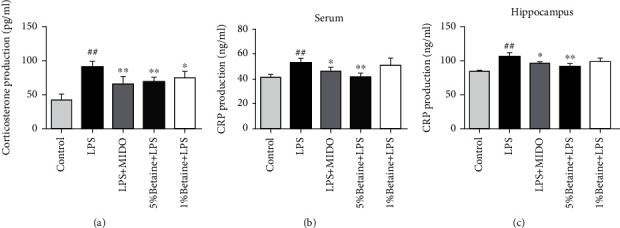
Determination of serum C-reactive protein (CRP) and corticosterone (CORT) levels in mice by ELISA. (a) Expression of corticosterone content in tissues. (b, c) Levels of C-protein in serum and hippocampus. ^##^*P* < 0.01 compared to the normal control group; ^∗^*P* < 0.05 and ^∗∗^*P* < 0.01 compared to the model group, *n* = 6.

The level of corticosterone in the hippocampus of mice was determined by ELISA, and the content of C-reactive protein in the serum and hippocampus was determined (Figures 5(a)–5(c)). The results showed that the levels of corticosterone and C-reactive protein in LPS-induced mice increased, and betaine significantly reduced the levels of serum C-reactive protein (CRP) and corticosterone (CORT) in LPS-induced mice.

### 5.5. The Effects of Betaine on LPS-Induced Expression of COX-2 and CD206 in Mice

**Figure 6:**
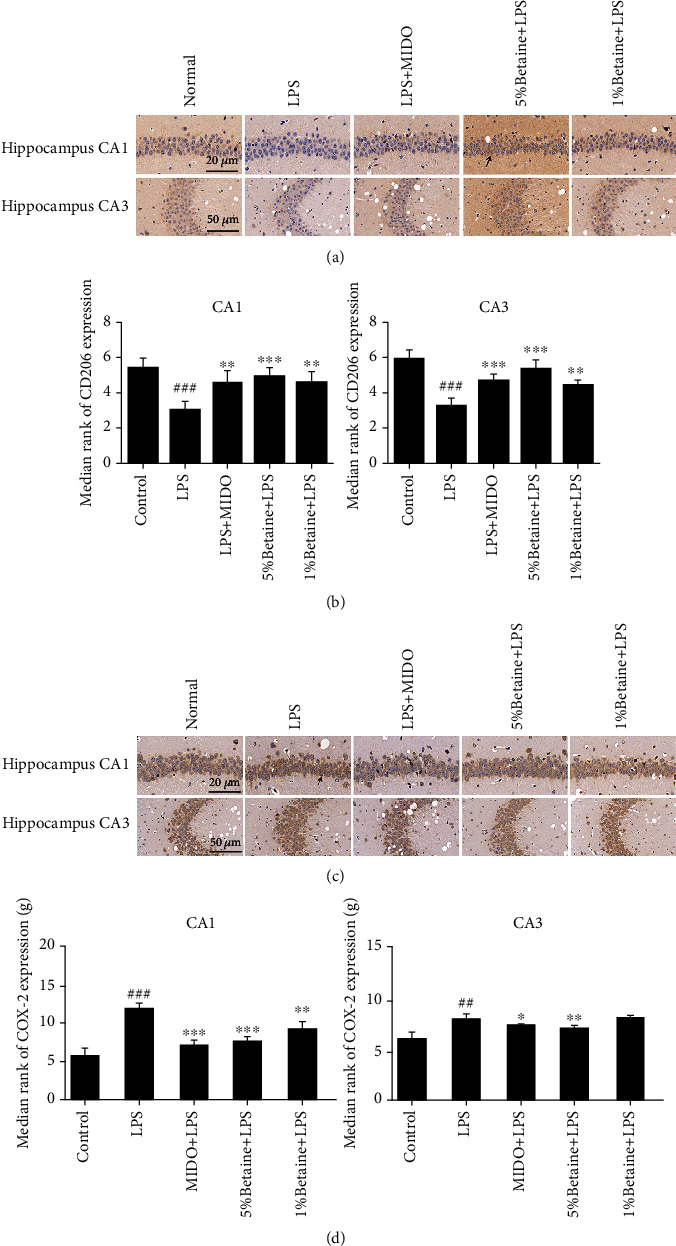
Immunohistochemical labeling of M1 polarization marker COX-2 and M2 polarization marker CD206 in mice CA1 and CA3 brain regions. Black arrows indicate positive immunohistochemical staining for M1/M2 polarization markers. (a) Typical immunohistochemical results for the effects of betaine on CD206 positive cells in mouse hippocampus. (b) Statistics on the number of CD206 positive cells. (c) Typical immunohistochemical results for the effects of betaine on COX-2 positive cells in mouse hippocampus. (d) Statistics on the number of COX-2 positive cells. The results were expressed as mean ± SEM; ^##^*P* < 0.01 and ^###^*P* < 0.001 compared to the normal control group; ^∗^*P* < 0.05, ^∗∗^*P* < 0.01, and ^∗∗∗^*P* < 0.001 compared to the model group, *n* = 6.

To determine whether the protective effect of betaine on lipopolysaccharide-stimulated microglia is related to polarization, we performed an immunohistochemistry. The M1 polarization marker COX-2 and the M2 polarization marker CD206 were labeled by immunohistochemistry in mouse brain regions (Figures 6(a) and 6(c)), and it was found that LPS could induce an increase in M1 polarization markers in mice (Figures 6(b) and 6(d)). Betaine and minocycline can reduce the expression of COX-2 to varying degrees and increase the expression of CD206, so it is suggested that betaine can promote the transformation of microglia from the M1 phenotype to the M2 phenotype.

### 5.6. The Effects of Betaine on the Expression of the NLRP3 Inflammasome in Mouse Brain Regions

**Figure 7:**
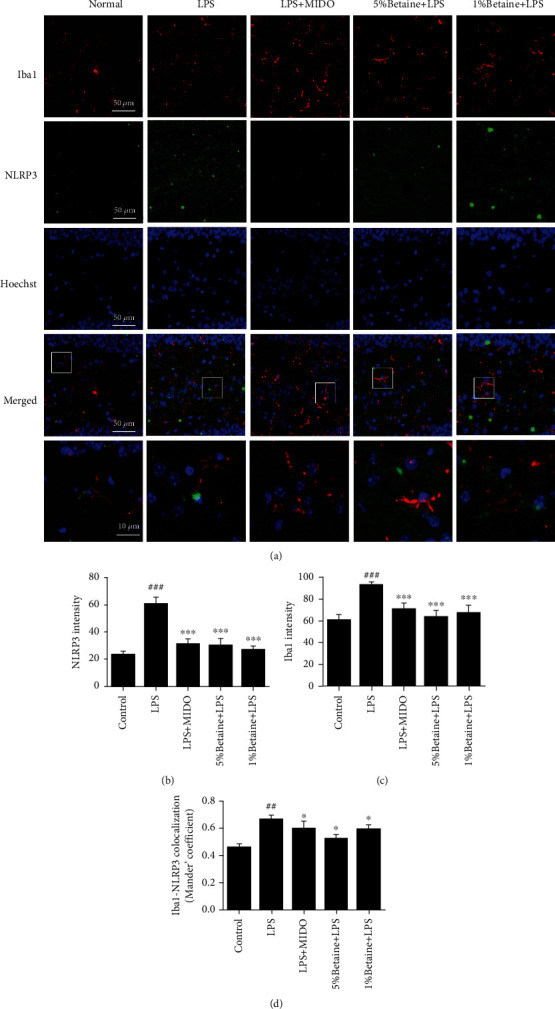
Betaine reduces the expression of NLRP3 inflammasome in mice GD brain region. Immunostaining with anti-NLRP3 (green) and anti-Iba1 (microglia activation marker, (red) antibodies, and nuclei stained with DAPI (blue). (a) Representative images of NLRP3 fluorescence intensity after stimulation with LPS. (b) Analysis of NLRP3 fluorescence intensity in brain regions. (c) Analysis of Iba1 fluorescence intensity in brain regions. (d) Colocalization analysis of NLRP3 and Iba1. The results were expressed as mean ± SEM; ^##^*P* < 0.01 and ^###^*P* < 0.001 compared to the normal control group; ^∗^*P* < 0.05 and ^∗∗∗^*P* < 0.001 compared to the model group, *n* = 6.

To determine whether the protective effect of betaine on lipopolysaccharide-stimulated microglia is related to NLRP3, we used an immunofluorescence assay. Iba1 is a microglial marker whose expression is positively correlated with microglial activation (Figure 7(a)), and the fluorescence intensity of Iba1 indicates the degree of microglial activation. The results showed that the expression of NLRP3 in the brain region of mice induced by LPS increased, the degree of activation of microglia increased, and the expression of NLRP3 in the brain region of mice treated with betaine decreased to varying degrees (Figures 7(b)–7(d)).

### 5.7. Effects of Betaine on LPS-Induced Mouse NLRP3/TLR4/NF-*κ*B Signaling and Microglial Polarization

**Figure 8:**
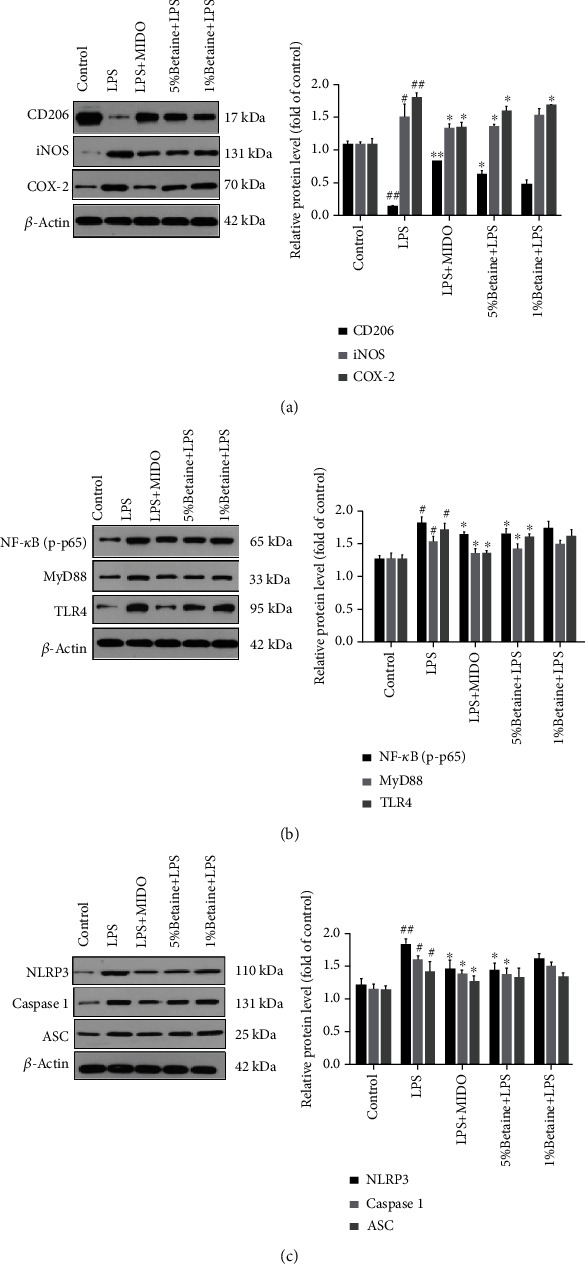
The effects of betaine on LPS-induced NLRP3/TLR4/NF-*κ*B signal transduction in mice. Western blot analysis of (a) CD206/iNOS/COX-2 protein levels, (b) P-NF-*κ*B/MyD88/TLR4 protein levels, and (c) NLRP3, caspase-1, and ASC protein levels; data were presented as mean ± SEM; ^#^*P* < 0.05 and ^##^*P* < 0.01 compared to the normal control group; ^∗^*P* < 0.05 and ^∗∗^*P* < 0.01 compared to the model group, *n* = 4.

TLR4 is the recognition receptor of LPS, while Myd88 is the junction protein of the LPS-induced inflammatory process. We found that both TLR4 and Myd88 levels were elevated in LPS-induced mice, and betaine administration reduced the expression of TLR4 and Myd88. NF-*κ*B induces NLRP3 mRNA expression, which is required for the formation of the inflammasome complex, so we determined the expression of NF-*κ*B and NLRP3 related proteins by western blotting (Figures 8(b) and 8(c)). The results showed that the expression of NF-*κ*B and NLRP3 related proteins was upregulated in LPS-induced mice, and the related protein expression of NF-*κ*B and NLRP3 was decreased by the administration of betaine. To observe the polarization of microglial cells, we detected INOS and COX-2 (Figure 8(a)), the polarization markers of microglial M1 and CD206, and the polarization marker of M2. The level of microglial polarization was observed to be biased toward the M2 phenotype.

## 6. Discussion

Depression is one of the most important mental illnesses and is closely related to inflammation. Under stress or pathological conditions, microglia secrete inflammatory mediators that disrupt the neuronal function and impair neurogenesis, increasing the vulnerability to stress and promoting the occurrence and development of depression [26, 27]. Betaine is a natural product with an anti-inflammatory and antioxidant activities. Previous studies have shown that betaine is mainly involved in the synthesis and metabolism of central monoamine transmitters as an osmotic regulator and methyl donor, thus playing an auxiliary role in the clinical treatment of depression [19, 28]. Betaine alone also has antidepressant-like effects [29, 30]. However, the mechanism by which betaine improves lipopolysaccharide-induced depression-like behavior is unclear. Therefore, we investigated the neuroprotective effect of betaine on LPS-induced depression-like behavior in mice and its mechanism of action.

With research advances in recent years, the important role of immune inflammation in the pathology of depression has gradually emerged [31–33]. The LPS-induced depression model is one of the classic acute depression models. As an immune stimulator, LPS can initiate the body's innate immune response, induce the secretion of various inflammatory cytokines, and further induce the central nervous system's immune response. This extensive immune inflammation can eventually lead to a series of depressive-like symptoms, such as anhedonia and reduced mobility [34]. Therefore, in this study, a model of acute inflammation induced by LPS was constructed.

In our study, it was found that compared with the LPS model group, the betaine group could alleviate LPS-induced depression-like behaviors, such as weight loss and decreased food intake, 24 hours after LPS injection. In our previous preliminary experiments, it was found that the dose of 5% betaine was the best effective dose. It was also confirmed in behavioral experiments, and it was found that high-dose betaine had a more effective inhibitory effect on LPS-induced depression-like behavior.

In many experimental animal models, minocycline has significant efficacy in attenuating central nervous system- (CNS-) induced neuroinflammation and depressive behavior and preventing lipopolysaccharide-induced cognitive impairment in mice [35]. Previous studies have reported that minocycline is a second-generation tetracycline that selectively inhibits microglial activation, and M1ibit activation of LPS regulates the polarization of microglia to the M2 phenotype and exert an anti-inflammatory effect. In the present experiment, minocycline had a protective effect on LPS-induced depression-like behavior in mice, and minocycline inhibited M1 microglial activation to protect against inflammation, which was in line with numerous studies.

The mechanism by which inflammation causes depressive-like behaviors likely involves one or more inflammatory molecules, such as C-reactive protein (CRP) or prostaglandin E2 (PGE2) and the hypothalamus–pituitary–adrenal (HPA) axis [36]. CRP is an acute phase response protein produced by the body after being stimulated by stress, and it is the most sensitive marker of the systemic inflammatory response. Under normal circumstances, the content is very small, and its blood concentration rises sharply during acute trauma and infection. We measured the content of CRP in the serum and hippocampus of different groups of mice 24 hours after LPS injection and found that betaine can reduce the expression level of CRP in mice and alleviate the inflammatory response of mice induced by LPS injection.

In terms of signaling, it is now recognized that the two most important systems involved in the stress response are the sympathetic branch of the autonomic nervous system and the hypothalamic–pituitary–adrenal (HPA) axis [37]. LPS induces oxidative stress and the release of proinflammatory factors, leading to corticotropin-releasing hormone (CCH), elevated serum corticosterone, hypothalamic–pituitary–adrenal axis dysfunction, and depression-like behaviors in animals. In this study, it was found that the CORT concentration in the hippocampus of the LPS model group mice increased, depression behaviors were increased, and these behaviors were reversed by betaine, which is consistent with the positive drug minocycline study results. Thus, these findings and the results of this experiment suggest that betaine reduces CORT concentrations and improves depressive-like behavior. These results further confirm that betaine has an anti-inflammatory activity, possibly related to antidepressant-like effects.

Neuroinflammation is a crucial pathological basis for depression, and various classical antidepressant treatments can improve neuroinflammation and exert antidepressant effects. Persistent inflammation accompanied by elevated levels of proinflammatory cytokines can lead to depressive symptoms [38], so the overproduction of proinflammatory cytokines plays a key role in the development and progression of psychiatric disorders. Proinflammatory cytokines, including interleukin-6 (IL-6) and tumor necrosis factor-alpha (TNF-*α*), have been found to be higher in patients with major depressive disorder [39], while their antioxidant capacity is lower [40]. Previous in vivo studies have reported that the inhibition of proinflammatory cytokines can alleviate depressive symptoms [41–43]. Furthermore, in mice, administration of IL-10 reduced the depression-like behaviors [44]. Therefore, modulating cytokine expression can alleviate depressive symptoms. In this experiment, betaine reduced the production of proinflammatory cytokines, including IL-6, IL-18, TNF-*α*, and IL-1*β* and increased the level of anti-inflammatory IL-10 compared with the LPS model group. Therefore, betaine can reduce LPS-induced systemic inflammation in mice. This is consistent with our expected results.

Overproduction of inflammatory cytokines and activation of microglia in the brain is implicated in depression and dysfunction in brain signaling. The hippocampus is a key brain region responsible for learning and memory and is closely related to the occurrence and development of depression. Microglia have both resting and activated states; in a healthy state, MGs are resting, and during CNS injury due to infection, brain injury, or ischemic injury, the microglial transition from a resting state amoeba is activated [45] and releases a large number of neurotoxic substances, such as oxygen free radicals and proinflammatory factors, causing an inflammatory response in the central nervous system. In addition to the resting state, there are two functionally distinct activated states, M1 and M2 [46]. The M1 microglial phenotype exerts a proinflammatory effect, while the M2 phenotype is involved in the anti-inflammatory processes in the brain. The M1 phenotype can be induced by LPS with increased production of proinflammatory cytokines. Most compounds that reduce neuroinflammation inhibit only microglia of the M1 phenotype. Few compounds promote microglial polarization to the M2 phenotype [7]. Therefore, how to inhibit the excessive activation of microglia to reduce the damage caused by the inflammatory response is particularly important. In our previous study, it was demonstrated that betaine induces polarization of microglia toward the M2 phenotype and inhibits LPS-induced inflammatory responses by inhibiting the TLR4/NF-*κ*B pathway. In our experiment, betaine significantly reduced the LPS-induced protein expression of iNOS and COX-2, which are related to M1 markers, in mouse hippocampal microglia and upregulated the protein expression of CD206, a M2 marker, in mouse hippocampal microglia. Therefore, betaine may modulate the polarized phenotype of microglia in LPS-induced depression model mice and induce the transformation of the microglial phenotype from M1 to M2 type.

Recent evidence suggests that the causative agent of depression may be associated with NLRP3 [3, 14]. NLRP3 inflammasome is activated by signals from the TLR4/MyD88/NF-*κ*B pathway that upregulates NLRP3 and pro-IL-1*β*, and from DAMPs/PAMPs like ROS and ATP that recruit ASC and caspase-1 to promote inflammasome assembly. The complex then synthesizes and secretes IL-1*β*, which triggers an inflammatory cascade [47–49]. Zhang et al. found that CUMS activated the NLRP3 inflammasome and increased IL-1*β* content in rat hippocampus, and administering the NLRP3 inhibitor VX765 significantly alleviated the neuroinflammation and depression-like behavior in the animals [50]. This is consistent with our findings. We demonstrate that betaine can significantly reduce the expression of TLR4/MyD88/NF-*κ* pathway signaling, and betaine can reduce the expression of NLRP3 and the levels of IL-1*β* and caspase-1 compared with the LPS model group. Therefore, betaine may attenuate LPS-induced depression-like behavior by inhibiting M1 microglia polarization and promote M2 microglia polarization by inhibiting NLRP3 signaling.

Our findings confirmed that betaine can reduce the expression of TLR4/MyD88/NF-*κ*B and polarize microglia toward the M2 phenotype by modulating the NLRP3 signaling pathway, thereby attenuating depression-like behaviors in LPS model mice. These results reveal the important role of the NLRP3 inflammasome in microglial activation and provide new ideas for the clinical application of betaine.

## 7. Conclusion

Betaine can effectively alleviate LPS-induced depression-like behavior in mice, which may regulate the transition from the M1 to M2 phenotype of microglial cells by inhibiting the overactivation of NLRP3 inflammasome.

## Data Availability

The analyzed data sets generated during the present study are available from the corresponding authors on reasonable request.
